# Left pulmonary artery sling repair without cardiopulmonary bypass: A case report

**DOI:** 10.1016/j.ijscr.2024.109692

**Published:** 2024-04-23

**Authors:** Dhama Shinta Susanti, Suprayitno Wardoyo, William Makdinata, Atya Shabrina Monika, Matthew Billy, Ameru Ulfalian

**Affiliations:** a Senior Consultant of Pediatric Cardiac Surgery Subdivision, Cipto Mangunkusumo Hospital, Jakarta, Indonesia; b Resident at Thoracic, Cardiac, and Vascular Surgery Division, Department of Surgery, Faculty of Medicine, Universitas Indonesia, Indonesia

**Keywords:** Cardiopulmonary bypass, Left pulmonary artery sling, Surgical repair, Tracheal compression

## Abstract

**Introduction and importance:**

Left pulmonary artery sling is an uncommon condition observed in infants. The severity of the condition is determined by the compression of the broncho-tracheal tree induced by the ring sling compression. The main goal of the treatment is to adjust the left pulmonary artery and eventually relieving the compression through surgery. The long-term outcome associated with the complexity of the anomalies.

**Case presentation:**

A nine-months old patient complained of worsening respiratory distress. The computed tomography scan revealed the potential presence of a left pulmonary artery sling and compression of the trachea, without any abnormalities in the trachea itself. Echocardiography study showed no intracardiac lesion. We successfully did left pulmonary artery transection and re-implantation to main pulmonary artery without cardiopulmonary bypass.

**Clinical discussion:**

Pulmonary artery sling commonly treated with reimplantation of the sling to its origin that usually required cardiopulmonary bypass machine. However, in our case we delivered it without the need of cardiopulmonary bypass. The outcome result turned excellent with echo post-operative showed confluent pulmonary arteries.

**Conclusion:**

The optimal approach to treating congenital pulmonary artery sling is through early surgical intervention in symptomatic patients. Following surgical repair devoid of tracheal lesion, the prognosis appears favorable, and routine follow-up is required to determine the long-term effects.

## Highlights

• Left pulmonary artery (LPA) sling is an uncommon condition observed in infants.
• This case shows LPA transection and reimplantation without cardiopulmonary bypass.
• The observed anomalies' complexity correlates with pulmonary artery sling outcomes.

## 1 Introduction

Left pulmonary artery (LPA) sling is an uncommon condition observed in infants. Vascular rings encompass only 1–3 % of all congenital heart disease. The pulmonary artery sling (PAS) was observed in 1 in 17,000 children of school age as its prevalence [1,2]. Previous studies have described successful repair with the need of cardiopulmonary bypass machine (CPB). A surgical approach with median sternotomy and CPB assistance were considered as first line approach to repair the anomaly, either in the presence or in the absence of coexisting cardiac anomalies [[3], [4], [5]]. In this case report, we successfully did LPA transection and re-implantation to main pulmonary artery without the need of cardiopulmonary bypass assistance [6]. This case report was written following SCARE criteria [7].

## 2 Presentation of case

**Fig. 1:**
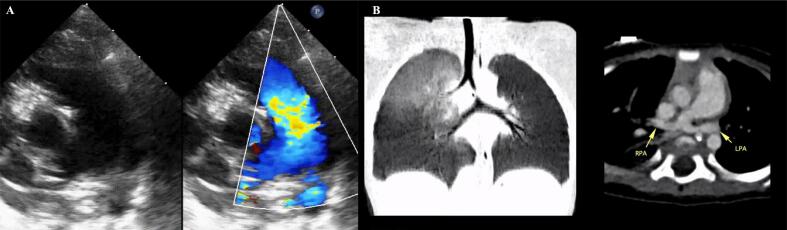
Preoperative assessment. Short axis view echo pre-operative showed LPA originated from RPA as shown in A. Pre-operative CT scan confirmed LPA as shown in B. The LPA can be seen originating from the RPA and wrapping around the distal trachea and right main stem bronchus en route to the left lung.

A nine-month-old girl was brought to our facility with dyspnea and recurrent upper respiratory infection for the past few months. Patient also presented with stridor and increasing work of breathing. The results of the CT scan confirmed that the LPA was originating from the right pulmonary artery (RPA) and passing posterior to the trachea, thereby causing tracheal compression without any abnormalities in the trachea itself. The pulmonary infection had resolved. Echocardiography revealed no congenital defects. Preoperative assessment was depicted in Fig. 1.

**Fig. 2:**
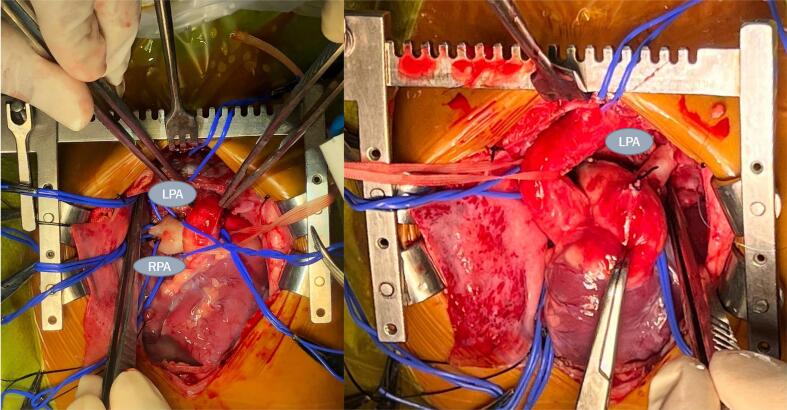
Intraoperative findings showed LPA originated from RPA. The LPA then re-implanted to MPA end-to-side, the anterior aspect was using interrupted suture, and the posterior was using continuous suture.

**Fig. 3:**
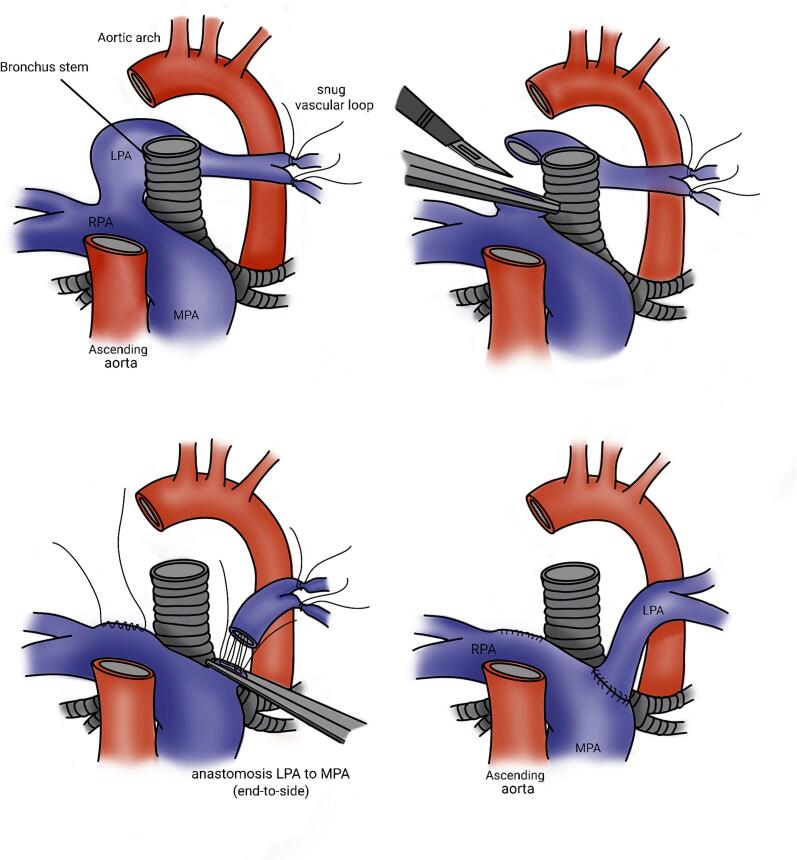
Intraoperative technique, the left PA was clamped and detached from its origin, the right PA, then was brought anterior to the trachea, and reimplanted to the main PA trunk without cardiopulmonary bypass support, without tracheoplasty.

A median sternotomy was utilized to perform the operation. An abnormal origin of the LPA was identified intraoperatively, originating from the RPA that passed behind the trachea. We also identified patent ductus arteriosus. The patent ductus arteriosus (PDA) was then doubly ligated and divided. Reimplantation of the left PA was performed in the absence of cardiopulmonary machine assistance. Without cardiopulmonary bypass support or tracheoplasty, the left PA was clamped and detached from its origin, and then the left PA was brought anterior to the trachea and reimplanted to the main PA trunk. Since RPA is patent, no significant desaturation was found while clamping and reimplanting the LPA. There were no notable tracheal anomalies detected throughout the procedure. Bleeding was controlled by reenforcing the suture. Intraoperative finding and step-by-step schematic illustration were depicted in Figs. 2 and 3 respectively. No complications were found during the procedure.

The patient was transferred to our cardiac intensive care unit following the operation. The primary respiratory issue was characterized by recurrent difficulty to wean off the ventilator. The patient kept having episode of desaturation and increased work of breathing while weaning from the ventilator. The result of partial pressure of carbon dioxide in arterial blood analysis was increased and arterial oxygen saturation was declined. Patient also had fever, with a peak of 38.5 °C. Chest X-ray post-operative confirmed hospital-acquired pneumonia and patient was treated with empiric antibiotic, cefoperazone 3 × 30 mg/kg and amikacin 1 × 15 mg/kg, according to our hospital microbial pattern. Culture of sputum showed *Klebsiella pneumoniae* which was sensitive to those two antibiotics. Apart from that, our patient received aspirin 5 mg per body weight single dose daily post-operative.

There was no major adverse event or complications after re-implantation of the LPA to MPA. Patient was hemodynamically stable and was successfully extubated on eighth day post-operative after weaning off slowly and administration of empiric antibiotic. The clinical condition was better, and patient was discharged at the eleventh post-operative day. There was no significant symptom at clinic and echocardiography performed revealed confluent pulmonary arteries, absence of pericardial effusion and dilatation, and normal biventricular function 14 days after the procedure.

## 3 Discussion

A PAS is an uncommon anomaly in which the left pulmonary artery originates abnormally from the posterior aspect of the right pulmonary artery. The anomalous left pulmonary artery ascends to the hilum of the left lung by traversing the right mainstem bronchus and then proceeding in a leftward direction, posterior to the trachea or carina and anterior to the esophagus. This causes symptoms in the upper airway by compressing the right mainstem bronchus and lower trachea [6].

Pulmonary artery sling commonly treated with reimplantation of the sling to its origin, which presently median sternotomy approach with CPB support, is the recommended method for surgical repair; however, in our case we were able to effectively restore the LPA without the CPB machine. The outcome result turned excellent with echo post-operative showed confluent pulmonary arteries. The benefits of CPB support include a motionless, blood-free operating field for the surgeon and adequate mobilization of associated vessels during surgery [8]. Nevertheless, the use of CPB causes the body to be exposed to extreme, non-physiologic conditions and initiates a global inflammatory response, resulting in damaging effects to the body, particularly in the case of infant. Due to their immature organ systems with altered homeostasis, reactive pulmonary vasculature, increased metabolic needs, and overall smaller stature, younger patients are more vulnerable to the inflammatory response to CPB [9]. A PAS repair without the need for a CPB machine might reduce the length of the procedure and protect pediatric patients from the harmful effects of CPB. Despite those benefits, repair without machine required higher technique skill and experience. Further research on necessity of using CPB on sling cases should be considered.

Medical treatment for patients with sling-bound pulmonary arteries, is supportive until surgical correction is possible. Ventilator-associated pulmonary artery sling patients who develop signs of airway obstruction and/or pneumonia may necessitate hospitalization. Infants may not require surgery if there is no airway obstruction, and the symptoms are mild. However, this is a very uncommon case. Following surgical repair, these symptoms of airway obstruction and/or pneumonia may persist, but they are expected to gradually resolve [6].

Anticoagulant treatment for pulmonary artery thrombosis following cardiovascular surgery in pediatric patients, as well as antithrombotic prophylaxis following PAS repair, lacks established guidelines at present. Our patient received aspirin 5 mg per body weight single dose daily post-operative. It is because more devastating if the patient had pulmonary thrombosis and embolism rather than bleeding since the risk of bleeding is minimal.

It appears that the complexity of frequently observed anomalies, such as complex cardiac and tracheal lesions, is correlated with the outcomes of PAS. The outcomes for children who do not require tracheal surgery and utilize a PA sling are remarkable. The requirement for tracheal surgery stipulates mortality [10].

## 4 Conclusion

Symptoms of pulmonary artery sling (PAS) may manifest as progressive respiratory distress. However, for treatment to be effective, a thorough pre-operative evaluation and surgical intervention are necessary. Following surgical repair devoid of tracheal lesion, the prognosis appears favorable, and routine follow-up is required to determine the long-term effects. The optimal approach to treating congenital pulmonary artery sling is through early surgical intervention in symptomatic patients.

## Informed consent statement

As the patient was a minor, written informed consent was obtained from the patient's parents/legal guardian for publication and any accompanying images. A copy of the written consent is available for review by the Editor-in-Chief of this journal on request.

## Ethical approval

Ethical approval is exempt/waived at our institution.

## Funding

There were no financial contributions made in support of the authors' research or authorship of this article.

## Author contribution

**Dhama Shinta Susanti**: Conceptualization, Funding acquisition, Investigation, Resources, Supervision, Writing – original draft, Writing – review & editing. **I Suprayitno Wardoyo**: Investigation, Supervision, Validation, Writing – review & editing. **William Makdinata**: Project administration, Resources, Supervision, Validation, Visualization, Writing – review & editing. **Atya Shabrina Monika**: Data curation, Visualization, Writing – original draft, Writing – review & editing. **Matthew Billy**: Data curation, Project administration, Visualization, Writing – original draft, Writing – review & editing. **Ameru Ulfalian**: Data curation, Investigation, Writing – review & editing.

## Guarantor

The senior author and guarantor of this manuscript is Dr. Dhama Shinta Susanti.

## Research registration number

Not applicable.

## Conflict of interest statement

The authors declared explicitly that there are no conflicts of interest with respect to the authorship and/or publication of this article.
